# Hypohydration alters pre-frontal cortex haemodynamics, but does not impair motor learning

**DOI:** 10.1007/s00221-022-06424-5

**Published:** 2022-07-26

**Authors:** Stephen P. J. Goodman, Maarten A. Immink, Frank E. Marino

**Affiliations:** 1 School of Allied Health, Exercise and Sport Science, Charles Sturt University, Bathurst, NSW Australia; 2 School of Science and Technology, University of New England, Armidale, NSW Australia; 3 Sport, Health, Physical Activity and Exercise Research Centre and College of Nursing and Health Sciences, Flinders University, Adelaide, SA Australia

**Keywords:** Cognitive performance, Dehydration, Human performance, Motor learning, Neuroimaging, Skill acquisition

## Abstract

It is unknown how hypohydration influences fine motor performance training and motor learning. Here, 30 participants (aged 19–46 years) were randomly assigned to a hypohydration (HYPO) or control (CON) group (both *n* = 15). Moderate hypohydration (~ 2.4% loss in body mass) was produced in HYPO via active dehydration before a 46 min fluid restricted rest period was undertaken. The conclusion of rest coincided with when CON attended the facilities. Both groups undertook a discrete sequence production task consisting of 6 training blocks, and returned ~ 300 min later to complete a delayed retention and transfer test while euhydrated. Bilateral pre-frontal cortex (PFC) haemodynamics were assessed using functional near-infrared spectroscopy throughout training and delayed learning assessments. Response time improved across training (*P* < 0.01) and was similar between the groups (both *P* = 0.22). Analysis of training PFC haemodynamics revealed a significant group by block interaction for oxygenated (O_2_Hb; *P* < 0.01), but not deoxygenated haemoglobin (*P* = 0.77). In training block 1, bilateral O_2_Hb was higher in HYPO (*P* = 0.02), while bilateral O_2_Hb increased in CON between blocks 2–3 and 5–6 (both *P* ≤ 0.03). During the delayed retention and transfer test, no group differences or interactions were found in response time, response error, or PFC haemodynamics (all *P* ≥ 0.27). Moderate hypohydration does increase PFC activation during motor skill learning, however, this appears to be transient and of little consequence to training or delayed retention or transfer performance.

**Supplementary Information:**

The online version contains supplementary material available at 10.1007/s00221-022-06424-5.

## Introduction

Water is essential for optimal physiological functioning. When undertaking exercise, or encountering heat stress, fluid loss may hinder thermoregulation (Cheuvront and Kenefick 2014). If poorly managed, water content of the fluid compartments can lead to a deficit (hypohydration) (Greenleaf 1992). Whilst research continues to explore the effects of hypohydration on exercise performance and physiological responses (Cheuvront and Kenefick 2014; Deshayes et al. 2020), the potential that changes in water content might have on cognition remains equivocal (Adan 2012; Wittbrodt and Millard-Stafford 2018). Despite this, Position Statements often indicate that moderate hypohydration (≥ 2% in body mass) is detrimental to cognitive performance (Sawka et al. 2007; Thomas et al. 2016). However, our own meta-analysis (Goodman et al. 2019a) alongside other contemporary empirical data (Goodman et al. 2019b; Irwin et al. 2017; MacLeod et al. 2018) are in contrast to this view. Whilst the implications hydration may hold for cognition continue to be debated, a key aspect of this construct; psychomotor performance is critical to athletes. Sporting success is unlikely to be decided through cognitive aspects alone, but rather may also be dependent on the ability to execute contextually appropriate skilled movements. Previously, Hillyer et al. (2015) in their narrative review into the effects of dehydration on skilled performance contend that such motor performances may be hindered by hypohydration specific alterations in cognitive (diminished perceptual mood or compromised attentional capacity) and physiologic (increased cardiovascular strain or altered glucose kinetics) function (Hillyer et al. 2015). Although, some studies suggest hypohydration is detrimental to skilled motor performance (Baker et al. 2007; Fortes et al. 2018; Lindseth et al. 2013; Smith et al. 2012), other empirical data demonstrate this physiological state may not impact basketball shooting (Dinu et al. 2018), motor racing performance (Mollica et al. 2019), or military target shooting and trigger control (Nolte et al. 2013). Despite the equivocal findings for motor performance, no study to date has investigated whether hydration may be of importance to developing the underlying procedural knowledge required for such performances; i.e. motor learning. This is surprising as hypohydration within the ranges currently believed to be detrimental to psychomotor performance, may actually arise during training (Duffield et al. 2012; Sawka et al. 2007; Thomas et al. 2016), when skills and/ or motor sequences (i.e. tactical plays) may be practiced or refined.

Several models have been proposed to account for the time-course of motor sequence learning (MSL) (Doyon et al. 2009; Hikosaka et al. 2002; Penhune and Steele 2012). Common among these is that learning is divided into phases. First, an early (fast learning) stage where rapid improvements in behavioural performance occur (Penhune and Steele 2012). Second, consolidation where performance becomes robust or asymptotic (Doyon et al. 2018; Penhune and Steele 2012) and third, an automatization phase (slow learning), where the sequence can be performed implicitly with little attentional requirement and/ or reactivation can occur (Doyon et al. 2009; Immink et al. 2020; Penhune and Steele 2012). Enabling this process are several neural structures including regions within the pre-frontal cortex (PFC), supplementary motor area (SMA), pre-motor cortex, primary motor cortex (M1), the associative and caudal regions of the basal ganglia, and the cerebellum (Doyon et al. 2009, 2018; Immink et al. 2020). Often two systems are discussed to account for MSL; the cortico-cerebellar and cortico-striatal systems. Within these, fast learning stimulates associative regions of the brain (i.e. associative striatum, PFC, cerebellar cortex) to begin establishing the novel motor routine (Doyon et al. 2009; Lohse et al. 2014). As practice continues, these associative areas begin to disengage, while sensorimotor structures within the corticio-striatal loop become more active, presumably to consolidate the motor representation (Doyon et al. 2018; Lohse et al. 2014).

How hypohydration influences the structure and function of the brain has been the subject of several neuroimaging investigations employing functional magnetic resonance imaging (fMRI) (Liu et al. 2013; Tan et al. 2019; Wittbrodt et al. 2018). Structurally, much of the aforementioned neuroanatomy involved with MSL are preserved when losses in body mass approximate to 3% (Tan et al. 2019; Wittbrodt et al. 2018). Functionally, Tan et al. (2019) investigated the M1 during a simple plantar flexion task, and found comparable blood oxygen level dependent (BOLD) responses during the task, irrespective of hydration status. Alternatively, during a rhythmic finger tapping visuomotor task, BOLD responses in several sites associated with motor learning (PFC, SMA, hippocampus, and the striatum) have been reported to be elevated during hypohydration (Wittbrodt et al. 2018). These authors also identified a decline in visuomotor accuracy over the 20 min task, and attributed this impairment to an inability of task-specific and non-task-specific regions of the brain to compensate despite elevated activation (Wittbrodt et al. 2018). Neuroimaging investigations have examined the effect of hydration on cognitive function and have predominantly incorporated fMRI, whereas, the present study utilises functional near-infrared spectroscopy (fNIRS). This non-invasive neuroimaging method incorporates near-infrared light at various wavelengths to distinguish concentrations of tissue oxygenated (O_2_Hb) and deoxygenated haemoglobin (HHb) (Thomas and Nam 2000). When cortical tissue is activated, variations in these chromophores arise, so that it can be used to quantify changes in neuronal function in response to task performance (Herold et al. 2018; Thomas and Nam 2000). Others (Hatakenaka et al. 2007; Immink et al. 2021; Ono et al. 2015) have examined the PFC during motor learning using fNIRS, however, the present study is the first to apply this methodology within the context of motor learning and hydration.

Given the dynamic interaction of neural systems shown to be involved with MSL, altering these neuronal synergies may have implications during the encoding process (fast learning and consolidation phases). Similarly, impaired motor performance during practice may also result in poorer acquisition. As hypohydration has the potential to alter both neurophysiologic function (Liu et al. 2013; Wittbrodt et al. 2018) and behavioural psychomotor performance (Baker et al. 2007; Smith et al. 2012), this physiological stressor may influence motor learning. The aim of this study was to investigate whether moderate hypohydration (~ 2% loss in body mass) would alter motor acquisition of a visuomotor task and determine whether this would have implications on delayed retention and learning transferability. To do this, we administered a conventional task used to evaluate motor training and delayed motor learning (discrete sequence production task; DSP task). Additionally, we examined the PFC throughout motor training and delayed learning assessments due to its involvement during the fast learning phase of MSL. We hypothesised that hypohydration would result in poorer motor sequence performance during motor training compared to euhydration. Additionally, we postulated that hypohydration would result in heightened PFC activity during the training phase of the DSP task. Lastly, we reasoned that those completing motor acquisition when hypohydrated would demonstrate poorer delayed retention and task transferability following a retention period, and that neural activity about the PFC would be higher during these tasks for this group.

## Materials and methods

### Participants

Study sample size was estimated a priori using G*Power v3.1.9.7 (Faul et al. 2007). The effect size used in this calculation was derived from the visuomotor response time data between the non-exercised control and moderately hypohydrated conditions in Wittbrodt et al. (2018); Cohen’s *f* = 0.17 (*d* = 0.34). Although the design of Wittbrodt et al. (2018) differs from the present study (within-subjects versus between-subjects), the approach used by these authors was the closest methodological representation, and provided the most conservative effect size estimate versus other studies incorporating independent group designs (*d* ranging from 0.48 to 4.32 Fortes et al. (2018) and Lindseth et al. (2013)). We powered the analysis to investigate the training phase of the study using a repeated-measures analysis of variance (ANOVA) with a within-between interaction. A sample of 30 was needed, assuming two groups, six measures, 0.80 power (β), an alpha of 0.05, correlation between measures = 0.62, and a non-sphericity ε = 1.

Thirty right-handed participants (20 males and 10 females; age = 33 ± 11 years) where recruited for the experiment. A total of 35 participants expressed interest in the study, but five were excluded because they did not satisfy the inclusion criteria; no evidence of pre-existing injuries or contraindicated conditions; not consuming any medications likely to influence the study (*n* = 2); were deemed right-handed (*n* = 3) according to a modified version of the Edinburgh Handedness Inventory (Milenkovic and Dragovic 2013). Included participants were pseudo-randomised into a control (CON) or hypohydration (HYPO) group, each consisting of 10 males and five females (descriptive data in Table 1). All females involved in the study were in the follicular phase of their respective menstrual cycle based on their self-reported history (6.3 ± 3.1 days). Prior to all testing sessions, participants were instructed to avoid strenuous physical activity, alcohol, and abstain from caffeine prior to attending the laboratories. All participants recruited for the study provided written informed consent for all study procedures that had been reviewed and approved by the University Human Ethics Committee (approval number 2016/040), and adhered with the requirements of the National Statement on Ethical Conduct in Human Research.

**Table 1:** Descriptive values of each group

Outcome	Control (*n* = 15)	Hypohydrated (*n* = 15)
Age (yrs)	31 ± 12	33 ± 10
Height (cm)	176 ± 11	176 ± 8
Mass (kg)	80 ± 20	79 ± 12
Peak oxygen consumption (mL/kg/min)	40.0 ± 6.9	42.3 ± 7.6

Data are presented as means ± SD

Note: 10 males and 5 females per group

### Procedure

Participants completed two sessions throughout the study. First, a familiarisation session where height (S + M Height Measure, Aaxis Pacific, Australia) and mass (HW-150 K, A&D Weighing, Australia) were obtained. Participants were also introduced to the DSP task that was used to quantify motor acquisition and learning, and a functional near-infrared spectroscopy (fNIRS) unit (Oxymon MKIII, Artinis Medical Systems, Netherlands) that was used to monitor cortical haemodynamics. Participants then completed a graded maximal cycling test to determine peak oxygen consumption, before completing 20 min of cycling (Ergomedic 834E, Monark, Sweden) in a climate chamber set at a hot and humid environment (climatic data not collected for familiarisation trials) to prepare them for if they were assigned to HYPO. Participants were then informed of their allocation, which had been determined a priori via coin flip and balanced for sex (pseudo-randomised) and instructed to consume at least two glasses of water to promote euhydration when reporting back to the laboratories to complete session two.

**Fig. 1:**
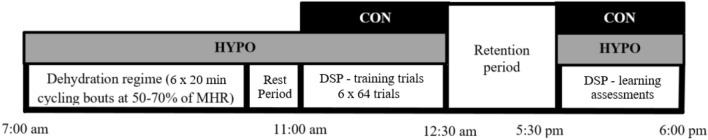
Overview of the study design. Participants were pseudo-randomised into either the control (CON; black) or hypohydrated (HYPO; grey) groups and arrived at the laboratory at the respective time indicated above. The dehydration regime, accompanying data collection, and the rest period took ~ 240 min (120 min of cycling, ~ 60 min of ancillary data collection/intermittent rest periods during cycling, and ~ 46 min of rest following the cessation of exercise). Both the control and hypohydration group completed the discrete sequence production (DSP) task training trials (~ 90 min) and engaged in a retention interval of ~ 300 min. Participants then returned to the facilities to perform the delayed learning assessments (retention and transfer test; ~ 30 min). MHR is maximal heart rate

Figure 1 shows a schematic of the procedures used in session two. Those assigned to HYPO arrived at the testing facilities at 7:00 am and provided a urine sample for determination of urine specific gravity (USG) by digital refractometry (PAL-10S, ATAGO®, Japan) to estimate hydration status. This urine marker is a measure of osmolality and increases when total body water is reduced (hypohydration; Cheuvront and Kenefick (2014). In the event, USG was greater than 1.020 mmol/L (*n* = 2, both in CON) the session was rescheduled. Participants then provided nude body mass (NBM) and affixed a sensor belt (EQ02 LifeMonitor, Equivital, United Kingdom) that transmitted heart rate (HR) and core temperature (*T*_C_) data via an ingestible telemetric sensor (VitalSense Core Body Temperature Capsule, Respironics Inc., USA) that was ingested at least 5 h before reporting to the facilities. A self-paced warm up was completed on the cycle ergometer and the participant then completed a dehydration regime modelled from Stewart et al. (2014) in a climate chamber (39.3 ± 1.4 °C, 36 ± 8% relative humidity (RH)). Briefly, this required participants to complete six cycling blocks lasting 20 min, whilst maintaining their HR between 50 and 70% of the maximum value attained from the maximal exercise test during familiarisation. Participants then exited the climate chamber, removed any affixed instruments, and provided NBM and a urine sample. A rest period (46 ± 16 min) in a normothermic environment (22.6 ± 2.4 °C, 65 ± 4% RH) was then undertaken to allow T_C_ to return to pre-exercise values, and minimise the confounding this and exercise may have had between the groups (Chang et al. 2012; Schmit et al. 2017).

The conclusion of the rest period coincided with the time CON arrived at the facilities (11:00 am). These participants provided a urine sample and had their body mass and T_C_ quantified as described above. All subsequent procedures of the study were identical between the groups. Both completed the training trials of the DSP task, exited the facilities and returned at 5:30 pm (retention interval of 302 ± 30 min) to complete a retention and transfer test. During the retention interval participants were instructed to consume fluid to promote euhydration upon returning to the facilities and to continue adherence to avoiding physical activity, caffeine, and alcohol. After their return, USG was collected to estimate hydration status and T_C_ was obtained.

### Motor sequence learning task

Motor sequence learning was investigated using a DSP task (Verwey, 1999) using the E-Prime experiment design platform (E-Prime® 2.0, Psychology Software Tools Inc., USA). Using a standard QWERTY keyboard, participants were trained on speeded and accurate production of four, 5-key sequences using their second to fifth digit (i.e. excluding the thumb) of the participants non-dominant (left) hand positioned over the V, B, N and M keys, which were numbered 1 to 4 from left to right (13,243, 21,324, 34,231, 42,312). Task instructions placed equal importance on accuracy and speed of completion for key press sequences. For all trials described below, participants were seated in front of a computer monitor in a normothermic room (21.4 ± 1.6 °C, 46 ± 7% RH) with their face positioned ~ 50–60 cm away from the screen. All cues, numbers and outcomes were presented to participants in 36 point, Times New Roman font, while the contents of the feedback screen was shown in 24 point.

**Fig. 2:**
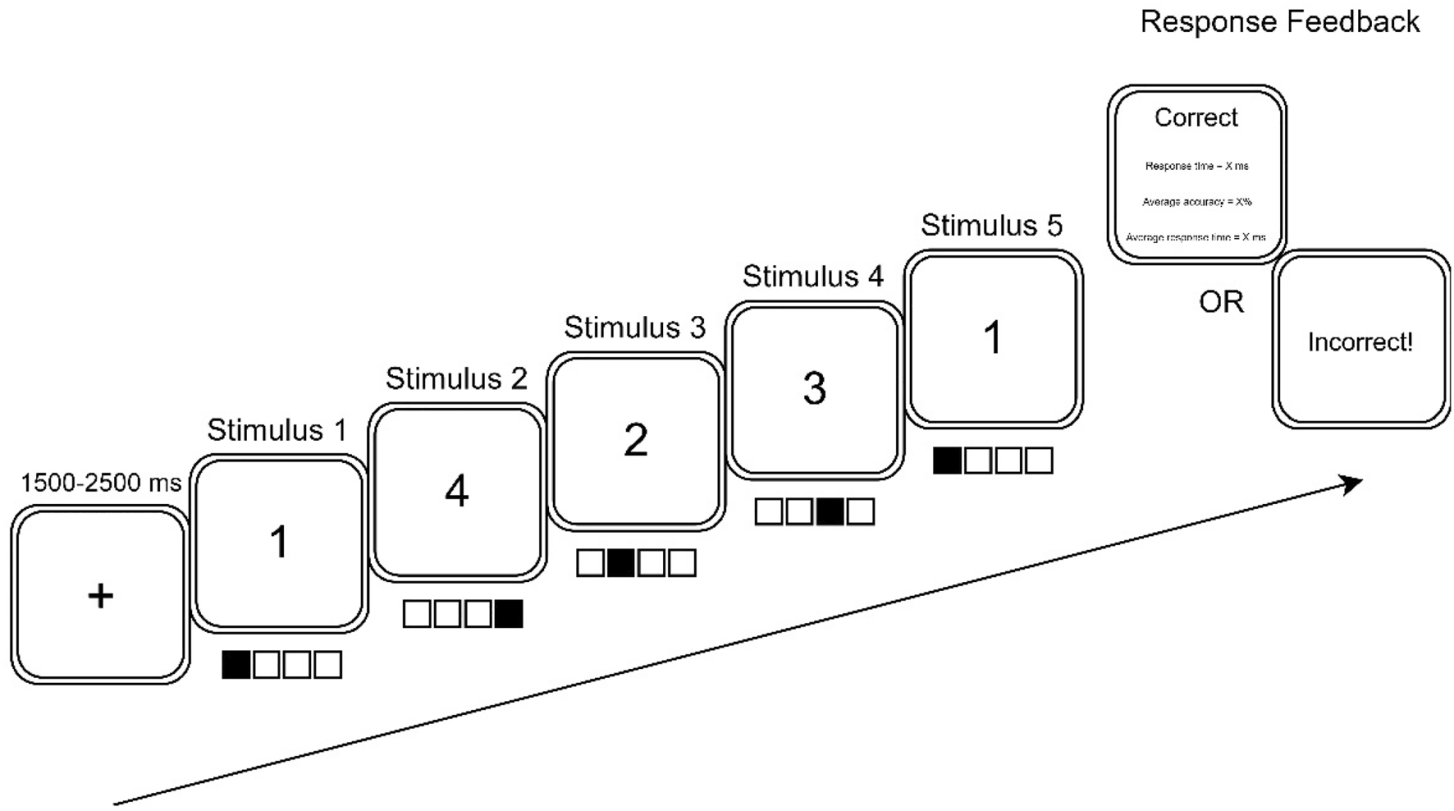
An example trial of the discrete sequence production task used in this study. Ascending numerical values corresponded to the second to fifth digit (i.e. excluding the thumb) on the participant’s non-dominant (left) hand. Values remained visible until a key was depressed or the final key press was made. Feedback was provided to the participant on their performance at the conclusion of each trial

Participants received six blocks of training on the four sequence variations, with a 60 s rest interval between blocks. Each block included 64 trials involving 16 trials per sequence variation. The order of sequence presentation followed a pseudo-random order with the criteria that a sequence would not be repeated on two consecutive trials. Figure 2 shows an example of how training trials were presented to participants. Initially, a fixation point appeared in the center of the screen for a random interval between 1500 and 2500 ms. Next, the fixation cross was replaced by a number representing the first key to be pressed. Upon, depression of the key, the next stimulus signalling the second keypress replaced the first stimulus and so on until the fifth and final key press stimulus was presented. If all five keypresses were pressed in the target sequence, then a feedback screen was presented to indicate response accuracy (i.e. correct), the response time to complete the 5-key sequence trial, and the running average of accuracy and response time for the block. If one or more keys were pressed incorrectly, an ‘incorrect’ message was displayed with no further performance feedback. In both cases, trial feedback was presented for 3000 ms.

Approximately 300 min after completion of the training session, delayed retention and transfer performance were assessed. Participants first completed retention evaluation based on one block of 20 trials that included 5 trials of each of the trained sequences in a pseudo-randomised order. A 60 s rest interval was then implemented, before a transfer test that introduced four novel 5 key sequences was undertaken (14,231, 23,143, 32,412, 41,324). Each transfer sequence was represented in 5 trials in a pseudo-random order within the 20 trial block.

### Functional near-infrared spectroscopy

**Fig. 3:**
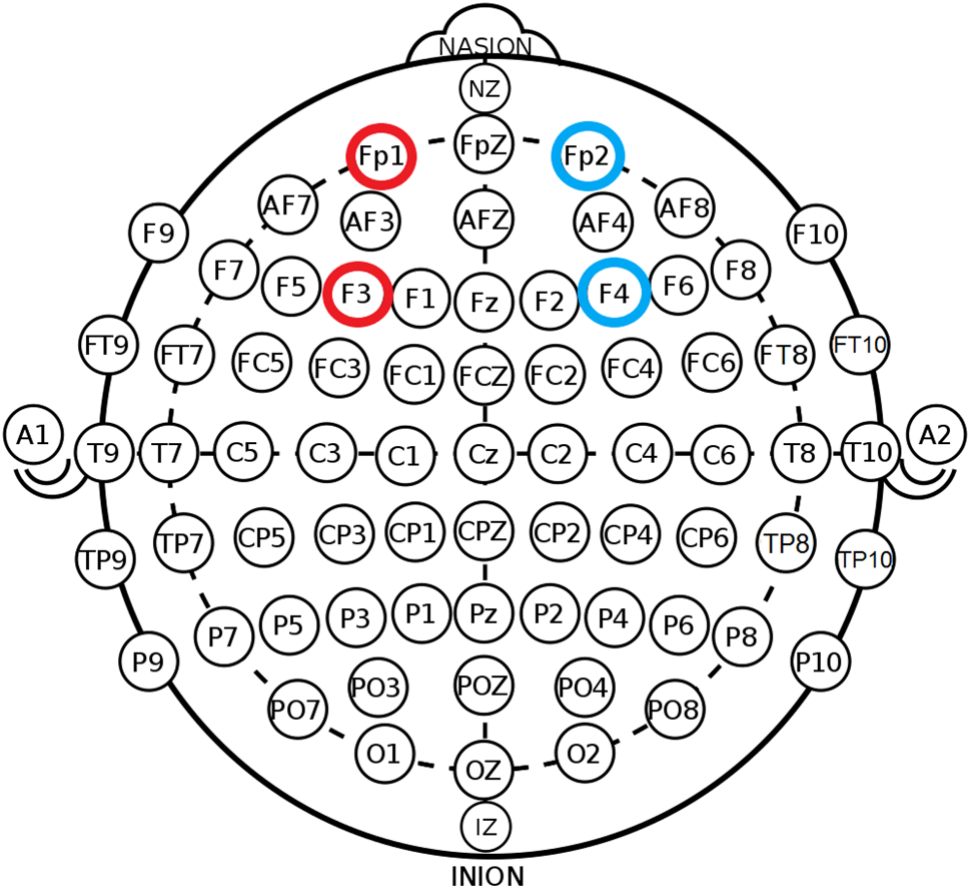
Functional near-infrared spectroscopy channel placement using the International 10–20 EEG system. Emitter-optode pairs were positioned over the left and right PFC, denoted between Fp1 and F3 (red), and Fp2 and F4 (blue) respectively. Original image was published under creative commons licence (CC0 1.0)

A 2-channel continuous-wave NIRS instrument was used to examine fluctuations in O_2_Hb and HHb cerebral haemoglobin concentrations. Due to the importance of the PFC in the fast learning phase of MSL (Doyon et al. 2018), probes were positioned bilaterally over this region. Specifically, an emitter-optode pair with an interoptode difference of 40 mm was positioned at the midpoint of Fp1 and F3, and Fp2 and F4 of the international EEG 10–20 system (Fig. 3), corresponding to the left and right PFC respectively (Perrey 2008). Other relevant neuroanatomy were not explored due to limitations in the technologies spatial resolution, and the availability of channels (only two at the time of data collection). The prospective sites were first prepared using an alcohol swab, and then the plastic casing securing the probes were secured to the skin using adhesive discs. The outline of each casing was then traced using a black marker to ensure common placement between the training and the retention and transfer test, before a black elastic wrap was applied to minimise extraneous light intrusion. All data were corrected using an age-dependent differential pathlength factor (Duncan et al. 1996) and sampled at 10 Hz. Placement sites and their corresponding real-time data were visually inspected for noise and indication of a heartbeat, as this is thought to represent acceptable optode-scalp coupling (Yücel et al. 2021). In the event a heartbeat could not be detected during inspection, the elastic wrap was loosened, adjustment was made to the channel, and the wrap was retightened before the data were again inspected. After an acceptable standard was attained, a 120 s eyes open baseline sample was collected, before participants commenced the training, or the retention and transfer test; separate baseline collections were obtained for each phase of the study. Markers were added throughout the training and delayed learning assessments to denote haemodynamic function during baseline collections, each training block, and the retention and transfer test. At the conclusion of the training period and the retention and transfer test, raw data were band-pass filtered in Oxysoft (Artinis Medical Systems, Netherlands) to minimise systemic noise and artefact. Cut-off frequencies were 0.5 and 0.01 Hz for low-pass and high-pass bands, respectively (Herold et al. 2018). Filtered data were then exported into a spreadsheet (Excel 2016, Microsoft, USA) for data analysis.

### Subjective estimates of performance

Hypohydration has the potential to alter perceptual mood state, and may compromise cognitive performance (Cheuvront and Kenefick 2014). Similar to those used by Armstrong et al. (2012), visual analogue scales (VAS) were used to examine perceptual states of attention, concentration, lethargy, and thirst. Each dimension was explored using a single VAS, and consisted of a 100 mm line with extreme positive and negative associations on either side. The following questions were asked in the VAS; “How alert are you right now?”, “How difficult is it to concentrate right now?”, “How tired do you feel right now?”, and “How thirsty are you right now?”. The VAS was administered to participants, prior to commencing the training trials of the DSP, at the conclusion of each training block (block 1–6), and prior to commencing the retention and transfer test.

### Peak oxygen consumption

The maximal exercise test completed during familiarisation was conducted using a cycle ergometer (Velotron Pro, RacerMate Inc., USA). The participant affixed a HR monitor (FT1, Polar, Finland), completed a brief self-paced warm up, and proceeded to commence the incremental test. Workload began at 100 W and increased incrementally by 20 W each minute. Throughout the test, cadence was required to be ≥ 60 RPM. Maximal HR was defined as the highest HR elicited. Respiratory measures were assessed breath-by-breath using an online system (True2400, ParvoMedics, USA) and averaged every 15 s. The peak oxygen uptake (VO_2PEAK_) was considered the highest value recorded by the system during the test.

### Data analysis

The magnitude of BM loss achieved in HYPO following the dehydration regime was determined using Eq. 1.

1 $$\left( {\frac{{Mass_{PRE} - Mass_{POST} }}{{Mass_{PRE} }}} \right) \times 100$$

Equation 1 Formula for deriving the magnitude of body mass reduction in the hypohydration group.

For the DSP training and delayed learning assessments, data were first inspected for response time outliers, defined as values that exceeded three standard deviations (SD) above the mean. Outliers were excluded from further analysis (0.02% of data). Then, mean percent accuracy and mean response time of accurate trials were calculated for each participant and block in each DSP task phase.

Pre-processed fNIRS data were first divided by laterality (right and left hemispheres), before epochs were generated for each baseline collection, training block, the retention, and transfer test. Each epoch was then examined for outliers, defined as three SD above the mean. Outliers were excluded from the analysis (1.16% of data). Epoch averages were then derived and the percentage change from the baseline collection was then determined by contrasting the averaged block, retention, or transfer test data to the respective baseline sample.

### Statistical analysis

All statistical analyses were conducted using the Statistical Package for the Social Sciences (SPSS, Chicago, Il, v27) and alpha was set at 0.05. Prior to statistical analysis, data normality was explored using the Shapiro-Wilks test. All data were normally distributed (*P* ≥ 0.07), except USG in CON prior to undertaking the retention and transfer test (*P* = 0.03), response error rate in block 2 in HYPO, block 4 in both, and the delayed learning assessments in both groups (all *P* = 0.01), response time in block 5 in CON (*P* = 0.02), O_2_Hb in blocks 2–6 for HYPO and block 6 in CON (all *P* ≤ 0.04), HHb in block 6 in HYPO, 1 and 3–6 and the transfer test in CON (all *P* ≤ 0.02), thirst in blocks 4–6 in CON and all thirst data for HYPO (all *P* ≤ 0.05), baseline concentration in CON (*P* = 0.03), attention at baseline and block 6 in CON and blocks 2, 3, and prior to the delayed learning assessments in HYPO (all *P* ≤ 0.04), baseline lethargy in CON, block 2 and prior to completing the retention and transfer test in HYPO (all *P* ≤ 0.02). Levene’s tests confirmed data homogeneity for all physiologic (all *P* ≥ 0.36), response error rate (all *P* ≥ 0.14), response time (all *P* ≥ 0.09), haemodynamic (all *P* ≥ 0.11), and subjective indices of performance (all *P* ≥ 0.06), except for the block 6 comparison for attention (*F*_(1,28)_ = 4.09; *P* = 0.05). A one-way ANOVA was used to examine physiologic data (USG and *T*_C_). During the training period, sequence learning was quantified using a mixed two-way ANOVA, applied to the response error rate and response time [all group (2; CON and HYPO) × block (6)]. Subjective estimates of performance (thirst, attention, concentration, and lethargy) were also analysed using a mixed ANOVA [all; group (2) × time (8; baseline, post-training block 1–6, and pre-learning assessment)]. For fNIRS, a three-way ANOVA [group (2) × laterality (2; left and right hemisphere) × block (7; baseline and block 1–6 epochs)] was used to analyse PFC haemodynamics (O_2_Hb and HHb) during training. When assessing retention and transfer test performance, a one-way ANOVA was applied to both response error rate and response time between the groups. Whilst, a three-way ANOVA [group (2) × laterality (2) × test (3; baseline, retention test epoch, and transfer test epoch) was applied to fNIRS data. A Bonferroni correction was used, and post-hoc testing was completed to explore significant interactions. Greenhouse–Geisser degrees of freedom corrections were applied as necessary when Mauchly’s test of Sphericity was violated. Effect sizes were represented using partial eta squared (η_P_^2^), where 0.04–0.24, 0.25–0.63, and ≥ 0.64 were considered the minimum practical, moderate, and strong effects, respectively (Ferguson, 2009). All data are expressed as means ± SD.

## Results

### Physiological outcomes

Table 2 shows the physiological data for each group throughout the respective intervention protocol. When arriving at the facilities, both the HYPO and CON were considered euhydrated (both USG ≤ 1.020 mmol/L). This criterion was surpassed for HYPO following the dehydration regime (Table 2). Prior to commencing the DSP training trials, T_C_ was similar between the groups (*P* = 0.59), while USG was greater in HYPO (*P* < 0.01). Following the retention interval, T_C_ and USG were similar between the groups before commencing the learning assessments (both *P* ≥ 0.50).

**Table 2:** Physiological outcomes for each group

	Control	Hypohydrated	*P*-value
Pre-exercise *T*_C_ (°C)	N/A	37.1 ± 0.4	
Pre-exercise USG (mmol/L)	N/A	1.014 ± 0.007	
Body mass loss (%)	N/A	2.4 ± 0.6	
Post-exercise *T*_C_ (°C)	N/A	38.4 ± 0.3	
T_C_ prior to training trials (°C)	37.0 ± 0.4	37.1 ± 0.5	0.59
USG prior to training trials (mmol/L)	1.012 ± 0.009	1.029 ± 0.003	< 0.01
T_C_ prior to retention and transfer tests (°C)	37.4 ± 0.3	37.3 ± 0.2	0.82
USG prior to retention and transfer tests (mmol/L)	1.012 ± 0.008	1.011 ± 0.006	0.50

Data are presented as means ± SD

Note: *T*_c_ is core temperature, USG is urine specific gravity, and *N/A* is not applicable

### Perceptual responses

Table 3 shows the subjective estimates of thirst, attention, concentration, and lethargy obtained throughout the study. Compared to CON, HYPO demonstrated greater thirst perception and lethargy (Both *F* ≥ 17.87; *P* < 0.01; β = 1.00), while attentiveness and concentration also declined during training (Both *F* ≥ 13.59; *P* < 0.01; β = 1.00). Main effects of time were also evident for all perceptual estimates (all *F* ≥ 19.60; *P* < 0.01; β = 1.00). Post-hoc analysis revealed poorer concentration in blocks 3–5 (all *P* ≤ 0.04), reduced attentiveness in blocks 3–6 (all *P* ≤ 0.04), and greater lethargy in blocks 3 and 4 (both *P* ≤ 0.02) compared to pre-DSP trial commencement. All perceptual responses were more favourable when participants returned to the laboratory to complete the learning assessments (all *P* < 0.01). Group × time interactions were found for each perceptual outcome (all *F* ≥ 5.76; *P* < 0.01; β ≥ 0.99). For thirst, attention, concentration, and lethargy, the HYPO group reported greater strain throughout all DSP training blocks (all *P* ≤ 0.02). Outcomes were similar between the groups when completing the learning assessments (all *P* ≥ 0.73). Post-hoc analyses also indicated that HYPO were less attentive in block 2 (*P* = 0.05) and more lethargic during blocks 2–4 (all *P* ≤ 0.05) compared to commencing the training trials of the DSP. For CON, compared to baseline, concentration and attentiveness declined during all the DSP training trials (all ≤ 0.02), and greater lethargy was apparent between blocks 2–6 (all *P* ≤ 0.05). Thirst perception did not increase for either group during training (all *P* ≥ 0.08).

**Table 3:** Subjective and perceptual data from the training and prior to the learning assessments

Outcome	Group	Discrete sequence production task—training period							Retention and transfer
		Baseline (pre)	Post-block 1	Post-block 2	Post-block 3	Post-block 4	Post-block 5	Post-block 6	Baseline (pre)
Thirst (a.u.)	Control	15.8 ± 12.3	16.0 ± 11.3	17.5 ± 10.2	20.8 ± 15.1	21.4 ± 15.4	21.5 ± 18.3	22.7 ± 20.1	6.3 ± 5.0^*^
	Hypohydration	74.1 ± 17.7^†^	73.7 ± 18.2^†^	77.1 ± 19.8^†^	77.1 ± 20.4^†^	79.1 ± 19.6^†^	78.1 ± 21.8^†^	78.7 ± 22.9^†^	6.4 ± 5.1^*^
Attention (a.u.)	Control	27.3 ± 25.6	35.3 ± 22.4^#^	38.8 ± 25.0^#^	43.3 ± 27.5^#^	48.1 ± 28.8^#^	46.6 ± 28.6^#^	48.5 ± 31.5^#^	6.1 ± 4.2^*^
	Hypohydration	67.3 ± 21.4^†^	73.1 ± 18.3^†^	76.7 ± 24.2^#†^	75.9 ± 24.9^†^	75.2 ± 23.8^†^	76.4 ± 22.7^†^	73.7 ± 22.6^†^	6.5 ± 7.5^*^
Concentration (a.u.)	Control	26.9 ± 24.6	34.1 ± 22.8^#^	37.5 ± 25.1^#^	40.3 ± 22.8^#^	45.0 ± 25.7^#^	45.5 ± 26.8^#^	45.5 ± 29.0^#^	6.5 ± 5.6^*^
	Hypohydration	68.1 ± 19.0^†^	72.1 ± 16.8^†^	73.6 ± 22.6^†^	76.0 ± 22.4^†^	72.9 ± 24.0^†^	73.9 ± 21.9^†^	72.8 ± 23.2^†^	7.0 ± 5.7^*^
Lethargy (a.u.)	Control	27.3 ± 23.4	33.2 ± 21.7	36.4 ± 25.8^#^	42.3 ± 26.1^#^	47.7 ± 27.9^#^	41.7 ± 28.0^#^	43.2 ± 30.2^#^	5.9 ± 5.8^*^
	Hypohydration	69.3 ± 24.1^†^	75.3 ± 21.1^†^	79.3 ± 26.3^#†^	78.6 ± 24.6^#†^	80.0 ± 23.8^#†^	79.1 ± 25.0^†^	77.0 ± 24.8^†^	5.0 ± 4.9^*^

Data are means ± SD

Note: a.u. are arbitrary units. #Denote differences in the respective group compared to the baseline sample (*P* ≤ 0.05). ^†^Denotes significant group by time interactions (*P* ≤ 0.03). *Indicates more favourable perceptual responses compared to data provided in the training trials (*P* ≤ 0.03)

### Motor sequence performance—Response error rate

Response error rate during training, retention, and the transfer test are reported in Table 4. There were no significant differences in error rate between the groups during training (*F*_(1,28)_ = 0.02; *P* = 0.88; β = 0.05). Additionally, there were no block (*F*_(5,140)_ = 0.44; *P* = 0.82; β = 0.19), or group × block (*F*_(5,140)_ = 0.32; *P* = 0.90; β = 0.17) interactions. No differences were found between the groups in the retention (*F*_(1,28)_ = 0.16; *P* = 0.69; β = 0.07) or transfer test (*F*_(1,28)_ = 0.23; *P* = 0.63; β = 0.08).

**Table 4:** Response error rate during training, retention, and the transfer tests

	Response error rate (%)							
	Block 1	Block 2	Block 3	Block 4	Block 5	Block 6	Retention	Transfer
Control	6.8 ± 3.2	6.6 ± 2.9	5.8 ± 3.7	5.7 ± 2.2	6.1 ± 3.6	5.6 ± 2.7	2.5 ± 3.03	3.9 ± 4.1
Hypohydration	5.9 ± 3.9	6.6 ± 5.6	6.4 ± 4.1	5.5 ± 5.1	6.7 ± 4.1	6.4 ± 2.4	3.0 ± 3.56	4.7 ± 4.3

Data are reported as means ± SD

Note: superior performance is indicated by a lower percentage of error

### Motor sequence performance—Response time

**Fig. 4:**
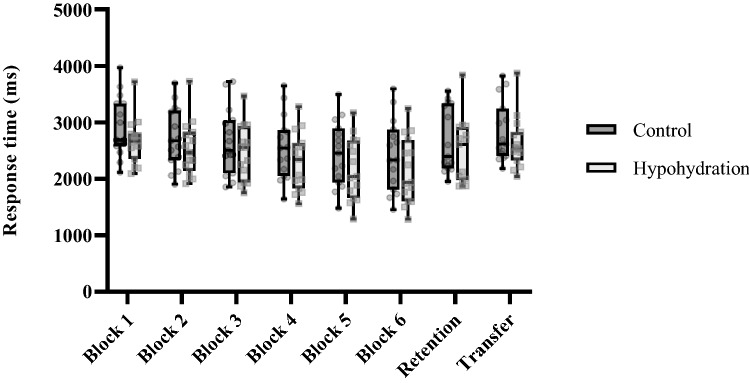
Response time during the training, retention, and the transfer test of the discrete sequence production task. Transparent circles and squares denote individual data for the control and hypohydration groups, respectively. A lower response time indicates faster performance

Response time during training, retention, and the transfer test are shown in Fig. 4. Throughout training, response time did not significantly differ between the groups (*F*_(1,28)_ = 1.54; *P* = 0.22; β = 0.23) and the group × block interaction was not significant (*F*_(2.5,70)_ = 0.52; *P* = 0.64; β = 1.00). A significant effect for block was observed (*F*_(2.5,70)_ = 72.15; *P* < 0.01; η_P_^2^ = 0.72; β = 1.00). Post-hoc analysis uncovered significant reductions in response time between block 1 and all other blocks (all *P* < 0.01), and between blocks 2–3, 3–4, and 4–5 (all *P* ≤ 0.03). No other blocks exhibited significantly different response time (all *P* = 1.00). Response time was similar between the groups in the retention (*F*_(1,28)_ = 0.98; *P* = 0.37; β = 0.16) and transfer test (*F*_(1,28)_ = 1.23; *P* = 0.27; β = 0.19).

### Near-infrared spectroscopy—training trails

**Fig. 5:**
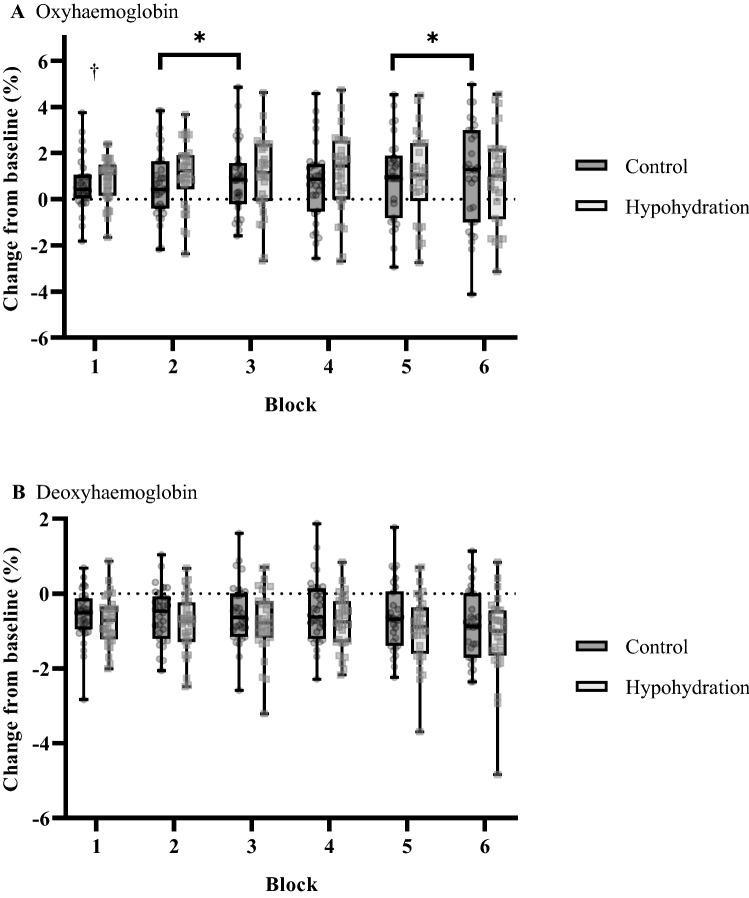
Changes in pre-frontal cortex **A** oxyhaemoglobin and **B** deoxyhaemoglobin concentrations evoked from baseline, during training. Transparent circles and squares denote individual data from either hemisphere for the control and hypohydration groups respectively. The *symbol represents significant pairwise comparisons for consecutive blocks in CON (both *P* ≤ 0.03). ^†^denotes a significant group × block interaction for HYPO (*P* = 0.02)

Figure 5 shows the changes in PFC O_2_Hb (Fig. 5A) and HHb (Fig. 5B) during the training trials. Between the groups O_2_Hb was similar (*F*_(1, 56)_ = 0.71; *P* = 0.40; β = 0.13). A significant interaction was found between group × block for O_2_Hb (*F*_(6, 51)_ = 3.52; *P* < 0.01; η_P_^2^ = 0.29; β = 0.92). Post-hoc analysis revealed O_2_Hb concentration was elevated in HYPO compared to CON during the initial training block (*P* = 0.02; Fig. 5A), while O_2_Hb increased between block 2–3 and 5–6 in CON (both *P* ≤ 0.03; Fig. 5A). An interaction was also found for laterality × block (*F*_(6, 51)_ = 2.61; *P* = 0.03; η_P_^2^ = 0.24; β = 0.81). Post-hoc analysis demonstrated elevated O_2_Hb of the right PFC between block 1 and 2 of practice (*P* = 0.02; Supplementary Appendix 1), no other blocks exhibited significantly elevated O_2_Hb concentrations (all *P* ≥ 0.14). A main effect of block was found for O_2_Hb (*F*_(6, 51)_ = 5.62; *P* < 0.01; η_P_^2^ = 0.13; β = 0.93). There was no interaction between group × laterality × block (*F*_(6, 51)_ = 0.50; *P* = 0.80; β = 0.19). HHb was similar between the groups (*F*_(1, 56)_ < 0.01; *P* = 0.97; β = 0.05). A main effect of block was exhibited for HHb (*F*_(6, 51)_ = 7.88; *P* < 0.01; η_P_^2^ = 0.49; β = 0.93). No significant group × block (*P* = 0.77; β = 0.19), laterality × block (*P* = 0.84; β = 0.22), or group × laterality × block (*P* = 0.14; β = 0.44), were found for HHb.

### Near-infrared spectroscopy—Retention and transfer test

**Fig. 6:**
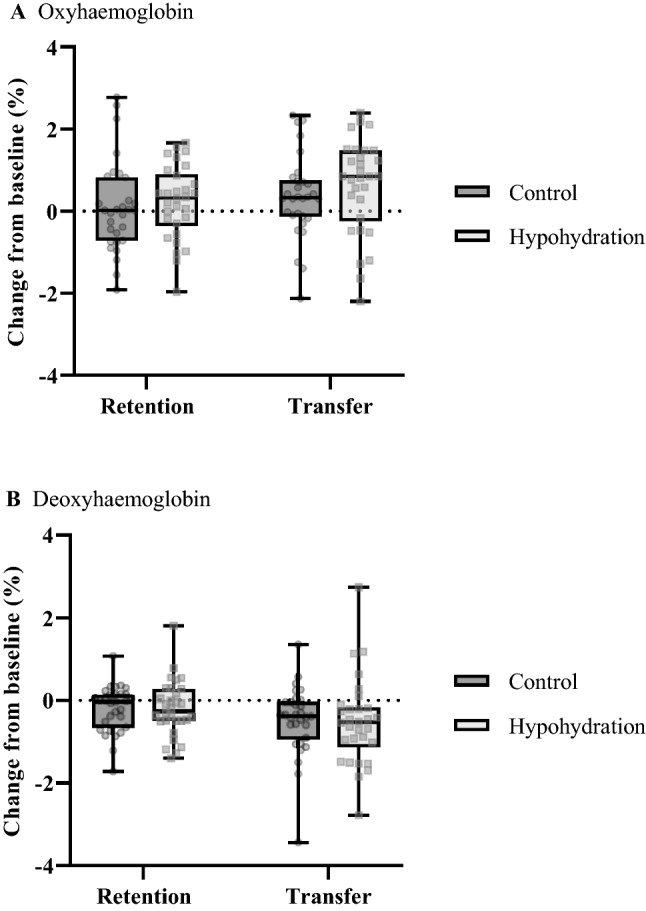
Changes in pre-frontal cortex **A** oxyhaemoglobin and **B** deoxyhaemoglobin concentrations evoked from baseline, during the retention and transfer tests. Transparent circles and squares denote individual data from either hemisphere for the control and hypohydration groups, respectively

Figure 6 shows the changes in the retention and transfer test. In each test, there were no significant differences in O_2_Hb or HHb between the groups (all *F* ≤ 1.26; *P* ≤ 0.95; β ≤ 0.20). A significant main effect for test was observed for O_2_Hb (*F*_(2, 55)_ = 9.41; *P* < 0.01; η_P_^2^ = 0.26; β = 0.97) and HHb (*F*_(2, 55)_ = 9.79; *P* < 0.01; η_P_^2^ = 0.26; β = 0.98). Post-hoc analysis revealed elevated O_2_Hb concentration during the transfer compared to the retention test (*P* < 0.01). A reduction in HHb concentration was also evident in the transfer compared to the retention test (*P* < 0.01). During the retention and transfer tests, no interactions were found for O_2_Hb or HHb for group × test (both *P* ≥ 0.58; β ≤ 0.13), laterality × test (both *P* ≥ 0.53; β ≤ 0.15), or group × laterality × test (both *P* ≥ 0.74; β ≤ 0.10).

## Discussion

The aim of this study was to investigate whether moderate hypohydration would alter motor acquisition of a visuomotor sequence learning task and whether this would have implications on delayed sequence retention and transferability. The dehydration regime resulted in a loss in body mass of ~ 2.4% in HYPO, however, motor sequence performance was similar between the groups. Divergent PFC responses were also found during acquisition, where O_2_Hb was elevated during the initial acquisition block and remained heightened in HYPO. Whereas for CON, O_2_Hb progressively increased throughout training. Novel to our study and contrary to our remaining hypotheses regarding the delayed learning assessments, undertaking motor acquisition whilst hypohydrated did not compromise sequence retention or transfer, and PFC haemodymics were comparable between the groups when executing these tasks whilst euhydrated.

### Motor sequence performance: training

As evidenced by the effects for block in response time during training, fast learning occurred in each group. Additionally, the extent of learning was similar irrespective of hydration status during training. Taken together, moderate hypohydration does not appear to influence motor sequence performance. Due to the novelty of our study, direct comparisons with the literature are difficult. Others (Cian et al. 2001; Pill et al. 2018; Wittbrodt et al. 2018) have explored the effects of hypohydration on acute instances of visuomotor task performance. Most akin to our design is the study of Wittbrodt et al. (2018) who monitored response time and accuracy during a 22 min paced visuomotor task in two euhydrated control conditions (one involving exercise and heat stress and one without), and following a dehydration regime that also produced moderate hypohydration (2.8 ± 0.3% of body mass). Although response time was similar among the conditions, Wittbrodt et al. (2018) report that more errors were made by the hypohydration condition compared to the non-exercising euhydrated control (69.7 ± 13.5% versus 85.1 ± 7.0%; *P* < 0.01). In contrast, we found no evidence that hypohydration impaired visuomotor response error rate or response time compared to the non-exercised control group. Although, the between group effects during training may have been underpowered to detect this finding, the group by block interactions during this period were suitably powered, and suggest no differences existed for these outcomes. Additional research is needed to clarify these findings.

### Pre-frontal cortex haemodynamics: training

MSL is thought to activate the frontal and associative regions of the brain as sequence representations form (Doyon et al. 2009). As consolidation occurs, activity of these regions decline, whilst cortico-striatal structures become pronounced (Doyon et al. 2018; Lohse et al. 2014). Our study examined PFC haemodynamics (O_2_Hb and HHb) during training via fNIRS. Heightened activity is thought to be the product of increased oxygen metabolism, and manifests as increased HHb and reduced O_2_Hb concentrations (Herold et al. 2018). Elevated cerebral activation may also lead to transient shifts in localised cerebral blood flow (neurovascular coupling), which alter haemodynamics and elevate the concentration of O_2_Hb and reduce HHb chromophores (Scholkmann et al. 2014). Interestingly, neither group demonstrated the disengagement previously reported, however, neurophysiologic responses were not similar between the groups. Indeed, CON demonstrated progressive increases in O_2_Hb throughout training. Whilst for HYPO, this chromophore was heightened in the initial training block and remained elevated throughout acquisition. Given HHb remained depressed throughout training in both groups this suggests divergent PFC activity patterns owing to neurovascular coupling (Scholkmann et al. 2014). Where, during practice the PFC became progressively more active in CON, while activation was heightened and remained so for HYPO.

One possible explanation for the PFC responses found in CON may be attributed to mental or cognitive fatigue. Mental fatigue is an altered psychophysiological state that has been shown to influence a range of cognitive and motor processes (Habay et al. 2021; Van Cutsem et al. 2017). In the present study, motor acquisition took 60–90 min, a range exceeding current thresholds thought to evoke this state (≥ 30 min; Van Cutsem et al. (2017)). Moreover, this group reported greater lethargy from block 2 onwards within the DSP task. Neuroanatomically, the frontal regions of the brain (such as the PFC) are thought to drive sustained cognitive performance (Ishii et al. 2014), and as shown by Wang et al. (2016), such regions may become more active to supplement behavioural task performance. It is possible, the PFC may have been compensating for the effects of cognitive fatigue, alongside development of sequence specific knowledge within striatal specific neuroanatomy. However, further research utilising imaging technologies with greater spatial resolution are needed to confirm this possibility.

For HYPO, PFC haemodynamics suggested elevated activation during the initial stages of training. Others (Kempton et al. 2011; Wittbrodt et al. 2018) have previously reported increased activity within task-specific neuroanatomy during instances of hypohydration. For the present study, the PFC operates alongside other neuroanatomical regions during the fast learning phase of MSL to establish the development of new motor routines (Doyon et al. 2009). Thus, our observation may reflect an additive task-specific activation that appears to occur alongside hypohydration and requires greater resource allocation for motor routine development. However, it is worth reiterating that our regression analysis revealed no relationship between altered cortical activity and improved motor sequence performance. Additionally, elevated neuronal function was sustained throughout motor acquisition. Collectively, these observations may suggest greater top-down control from frontal neuroanatomy was required during hypohydration, possibly to compensate for elevated physiologic or perceptual strain. Similar to CON, mental fatigue may also have been experienced, or even exacerbated due to heightened thirst (Goodman and Marino 2021). However, such additions likely remained within capacity, and may even have been ergogenic for sequence acquisition (Borragan, Slama, Destrebecqz, & Peigneux, 2016). More research is required to explore these themes.

An effect for laterality was also identified within the groups during training, where O_2_Hb was heightened within the right PFC during the initial two blocks of practice; suggesting heightened activation (Scholkmann et al. 2014). Foremost, it is worth reiterating the unilateral nature of the task used here. Participants completed all trials using their non-dominant (left) hand. Given the contralateral motor control required for such performances, it is logical to expect the right PFC to have been more active, due to its role in motor routine development and subsequent downstream descending command (Doyon et al. 2009). Additionally, Immink et al. (2021) have recently recounted the importance of the right PFC within the frontoparietal motor learning network (extending to the right SMA, pre-motor cortex, M1, and posterior parietal cortex). These authors examined the effects of interleaved and repetitive (blocked) practice on bilateral motor sequence learning, finding that repetitive practice resulted in greater PFC activation during a delayed retention test (Immink et al. 2021). The authors reasoned that the low contextual interference encountered by the interleaved practice group enabled more favourable motor learning to occur and promoted more efficient PFC activation during the retention test compared to the repetitive practice group (Immink et al. 2021). Lin et al. (2011) have also compared the effects of interleaved and repetitive practice on cortical function using fMRI, and report greater BOLD responses among key structures within the motor learning network (right pre-frontal and pre-motor cortices). In the present study, sequence trials were completed in a pseudo-randomised order with the condition that the same sequence would not be completed in consecutive trials. Therefore, our design is likely to have been aligned to interleaved practice. Given the findings discussed above, this may also explain why heightened activation about the right PFC was observed during training in the present study.

### Delayed assessments of motor learning: retention, transfer, and pre-frontal cortex haemodynamics

Behavioural performance, subjective indices, and neurophysiological responses were similar between the groups when undertaking delayed motor sequence retention and transferability. Likely, this was attributed to the comparable degree of motor training performed by the groups. However, a supplemental consideration is the shift in hydration status that occurred in HYPO throughout the retention interval. Previously, Edmonds et al. (2019) have proposed that memory functionality may be enhanced when shifting from a hypohydrated to euhydrated state. Evidently, motor learning as a form of procedural memory may have benefited from this physiological transition. However, in the event this should be ergogenic, one might expect the HYPO group to have performed superiorly in the retention and transfer tests, yet this was not the case. As such, the postulations of Edmonds et al. (2019) may be localised to more acute or cognitive applications of memory function (i.e. cued/ free recall settings), or may not be of benefit to off-line motor memory consolidation. Further research should be conducted to examine whether transitions in hydration state could be implemented strategically and be of benefit to a learner undertaking skill acquisition.

## Limitations and future research

A limitation of the present study is that prior to commencing the training trials of the DSP task, identical testing procedures were not undertaken by the groups. Indeed, HYPO performed a dehydration regime involving intermittent moderate exercise in the heat, whilst CON did not undertake heat stress or exercise. Previously, Chang et al. (2012) have identified meta-analytically that exercise produces an ergogenic effect on cognition for up to 20 min after its cessation, whilst Schmit et al. (2017) suggest cognitive performance is enhanced when *T*_C_ rises to ~ 38.5 °C. We attempted to control for the possible confounding effects of heat stress and exercise by implementing a rest period following the dehydration regime. This period exceeded the duration thought to benefit cognitive function by ~ 26 min (Chang et al. 2012) and was sufficient to allow T_C_ to return to pre-exercise levels (Table 2). Although we have attempted to account for the divergent pre-acquisition period, this should not exclude the possibility that the HYPO group may have been more advantageously placed to engage in motor acquisition. Future researchers should attempt to ensure study design enables homogenous pre-acquisition protocols. Additionally, only the PFC was examined in the present study. As already discussed, MSL involves several neuroanatomical structures. Whilst gold standard neuroimaging technologies (i.e. fMRI) have contributed to our detailed understanding of MSL and its time-course, such approaches prohibit investigations into many functional movement scenarios. Employing more flexible technologies such as fNIRS could be considered by future researchers. Although currently such approaches do not provide the spatial insights into key structures associated with consolidation (i.e. striatal regions; Lohse et al. (2014)), with ample probes, haemodynamic function can be explored in cortical structures such as the PFC, SMA, PMC, and M1, and may assist in better understanding skill acquisition in more ecologically valid contexts.

## Conclusion

This study investigated whether moderate hypohydration would impair motor sequence acquisition, and whether this would influence delayed sequence retention and transferability. The novel finding of this study is that moderate hypohydration (~ 2.4%) does increase bilateral PFC activity during motor skill learning. However, this does not compromise motor sequence learning performance compared to euhydration. Delayed motor sequence retention and transfer performance, and bilateral PFC function are similar when euhydration is restored.

## Supplementary Information

Below is the link to the electronic supplementary material.

Supplementary file1 (DOCX 101 KB)

## Data Availability

The datasets generated during and/or analysed during the current study are available from the corresponding author on reasonable request.
