# Supplementary material for: Hypohydration alters pre-frontal cortex haemodynamics, but does not impair motor learning
Source: Exp Brain Res. 2022 Jul 26;240(9):2255–68. doi: 10.1007/s00221-022-06424-5 (PMC9458583; doi:10.1007/s00221-022-06424-5)
Supplement: Supplementary file 1 — Supplementary file1 (DOCX 101 KB) [file 221_2022_6424_MOESM1_ESM.docx]

**Appendix 1.** Changes in pre-frontal cortex oxyhaemoglobin concentrations evoked from baseline between hemispheres. Transparent circles and squares denote individual data from either hemisphere for the control and hypohydration groups respectively. The * symbol represents significant pairwise comparisons for consecutive blocks in the right hemisphere (both *P =* 0.02).
